# GlcNAc6ST2/CHST4 Is Essential for the Synthesis of R-10G-Reactive Keratan Sulfate/Sulfated *N*-Acetyllactosamine Oligosaccharides in Mouse Pleural Mesothelium

**DOI:** 10.3390/molecules29040764

**Published:** 2024-02-07

**Authors:** Yoshiko Takeda-Uchimura, Midori Ikezaki, Tomoya O. Akama, Yoshito Ihara, Fabrice Allain, Kazuchika Nishitsuji, Kenji Uchimura

**Affiliations:** 1 Univ. Lille, CNRS, UMR 8576—UGSF—Unité de Glycobiologie Structurale et Fonctionnelle, F-59000 Lille, France; yoshiko.uchimura@univ-lille.fr (Y.T.-U.); fabrice.allain@univ-lille.fr (F.A.); or nishit@wakayama-med.ac.jp (K.N.); 2 Department of Biochemistry, School of Medicine, Wakayama Medical University, Wakayama 641-8509, Japan; ikezaki@wakayama-med.ac.jp (M.I.); y-ihara@wakayama-med.ac.jp (Y.I.); 3 Department of Pharmacology, Kansai Medical University, Osaka 570-8506, Japan; akamat@hirakata.kmu.ac.jp

**Keywords:** sulfotransferase, keratan sulfate, sialomucin, mesothelium, MUC16

## Abstract

We recently showed that 6-sulfo sialyl *N*-acetyllactosamine (LacNAc) in *O*-linked glycans recognized by the CL40 antibody is abundant in the pleural mesothelium under physiological conditions and that these glycans undergo complementary synthesis by GlcNAc6ST2 (encoded by *Chst4*) and GlcNAc6ST3 (encoded by *Chst5*) in mice. GlcNAc6ST3 is essential for the synthesis of R-10G-positive keratan sulfate (KS) in the brain. The predicted minimum epitope of the R-10G antibody is a dimeric asialo 6-sulfo LacNAc. Whether R-10G-reactive KS/sulfated LacNAc oligosaccharides are also present in the pleural mesothelium was unknown. The question of which GlcNAc6STs are responsible for R-10G-reactive glycans was an additional issue to be clarified. Here, we show that R-10G-reactive glycans are as abundant in the pulmonary pleura as CL40-reactive glycans and that GlcNAc6ST3 is only partially involved in the synthesis of these pleural R-10G glycans, unlike in the adult brain. Unexpectedly, GlcNAc6ST2 is essential for the synthesis of R-10G-positive KS/sulfated LacNAc oligosaccharides in the lung pleura. The type of GlcNAc6ST and the magnitude of its contribution to KS glycan synthesis varied among tissues in vivo. We show that GlcNAc6ST2 is required and sufficient for R-10G-reactive KS synthesis in the lung pleura. Interestingly, R-10G immunoreactivity in KSGal6ST (encoded by *Chst1*) and C6ST1 (encoded by *Chst3*) double-deficient mouse lungs was markedly increased. MUC16, a mucin molecule, was shown to be a candidate carrier protein for pleural R-10G-reactive glycans. These results suggest that R-10G-reactive KS/sulfated LacNAc oligosaccharides may play a role in mesothelial cell proliferation and differentiation. Further elucidation of the functions of sulfated glycans synthesized by GlcNAc6ST2 and GlcNAc6ST3, such as R-10G and CL40 glycans, in pathological conditions may lead to a better understanding of the underlying mechanisms of the physiopathology of the lung mesothelium.

## 1. Introduction

Five human members and four of their mouse orthologs represent the GlcNAc-6-*O*-sulfotranseferase (GlcNAc6ST) family [1] (Table 1). GlcNAc6ST catalyzes the transfer of the sulfuryl moiety (-SO_3_, abbreviated as “sulfate” or “S”) to position 6 of the GlcNAc residues of sulfated glycans such as keratan sulfate (KS) and L-selectin ligand sialomucins. The expression patterns of these enzymes vary among tissues, and syntheses of sulfated glycans and glycan epitopes mediated by these enzymes are occasionally common and sometimes distinct. These biosynthetic reactions are strictly regulated by physiological and pathological conditions. It is important to determine which GlcNAc6STs are involved in the synthesis of which sulfated glycans in a tissue-specific and cell-specific manner. Recently, we reported that sialyl 6-sulfo *N*-acetyllactosamine (sialyl 6-sulfo LacNAc, Neu5Acα2-3Galβ1-4(6S)GlcNAc), a sialylated GlcNAc-6-sulfated glycan recognized by the CL40 antibody [2], is present in the mesothelin-positive mesothelium of pulmonary pleura under physiological conditions [3]. We also reported that the synthesis of CL40-positive glycans in the mouse lung mesothelium requires GlcNAc6ST2 (encoded by *Chst4*) and GlcNAc6ST3 (encoded by *Chst5*) but not GlcNAc6ST1 (encoded by *Chst2*) or GlcNAc6ST4 (encoded by *Chst7*) [3]. GlcNAc6ST2 and GlcNAc6ST3 are complementary to the synthesis of CL40-reactive sialylated sulfated glycans in the mouse pleural mesothelium. Since we previously showed that the synthesis of cerebral R-10G-positive KS in which the predicted minimum epitope is an asialo 6-sulfo di-LacNAc [4,5,6,7] is GlcNAc6ST-dependent and that GlcNAc6ST3 is a major GlcNAc6ST in the adult brain [8,9,10], we tested the possibility that R-10G-reactive KS is also present in the pleural mesothelium. The question of which GlcNAc6STs are responsible for the synthesis of R-10G-reactive glycans was elucidated as well. Here, we found that R-10G-reactive glycans are abundant in the pulmonary pleura, similar to the observation of CL40-reactive glycans, and that GlcNAc6ST3 is only partially involved in the synthesis of R-10G glycans in the pleura, unlike in the adult brain. Remarkably, GlcNAc6ST2 is essential for the synthesis of R-10G-positive KS/sulfated LacNAc oligosaccharides in the pleura. The type of GlcNAc6ST involved in KS glycan biosynthesis and the extent of its contribution to the biosynthesis were found to differ among tissues in vivo. We also showed that the GlcNAc6ST2 is sufficient for the synthesis of R-10G-reactive glycans in the pulmonary pleura. In KSGal6ST (encoded by *Chst1*) and C6ST1 (encoded by *Chst3*) double-deficient (DKO) mice [11], the level of R-10G immunoreactivity in the pleura was clearly increased. MUC16, a mucin molecule, was shown to be a candidate carrier protein of the R-10G-reactive glycan.

## 2. Results

### 2.1. R10G-Reactive Sulfated Glycans Are Abundant in the Mouse Pleural Mesothelium

**Figure 1:**
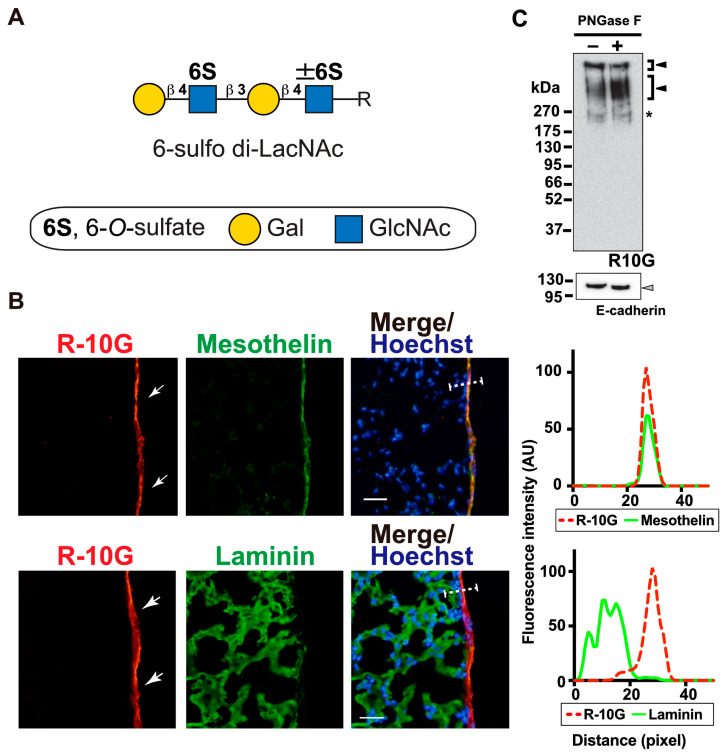
R10G-reactive sulfated glycans are present in the mouse pleural mesothelium. (**A**) Schematic representation of 6-sulfo di-LacNAc recognized by R-10G. C-6 sulfate (S), galactose (Gal), and *N*-acetylglucosamine (GlcNAc) are shown. The glycan is extended from variable underlying core glycans (R). (**B**) Sections of lungs from normal adult mice were co-stained with R-10G (red) and an anti-mesothelin antibody (upper, green) or an anti-laminin (lower, green), followed by Hoechst 33342 nuclear staining (blue). Shown are representative fluorescence microscopy images of the lower and central portions of the left lung lobe (*n* = 3). Dense R-10G staining signals in the pleural mesothelium (arrows) revealed by co-staining with the mesothelial marker mesothelin are presented. Plot profiles of R-10G and mesothelin staining or laminin staining are presented. Signal intensities along the line marker (white dashed line) paths in the merged images were determined. Scale bar: 20 µm. (**C**) GlcNAc-containing fractions of mouse lung lobes were obtained with wheat germ agglutinin-coated beads. The bead-bound materials were incubated without or with PNGase F. The immunoreactivity of R-10G was tested. Bands with molecular weights of >270 kDa were observed (closed arrowheads). E-cadherin was used to show protein equal loading and the successful pretreatment of PNGase F of the lung fraction. The 110 kDa band shifted from 120 kDa in the pretreated fraction (gray arrowhead). Bands with 240 kDa were also seen in IgG1 control blots (asterisk) [3].

R-10G recognizes KS and related glycans [4,8,9]. The minimum recognition determinant of R-10G is 6-sulfo di-LacNAc, Galβ1–4GlcNAc(6S)β1–3Galβ1–4GlcNAc(±6S) (Figure 1A). We examined whether R-10G-reactive KS glycans are present in a steady state in mouse lung pleurae. We found strong R-10G immunoreactivity in the pleural mesothelium (Figure 1B). These staining signals co-localized with those of an antibody to mesothelin, a marker of mesothelial cells that line the lung pleura, but differed from those of an antibody to laminin, a marker of the basement membranes of the alveolar epithelium, endothelium, and visceral pleura (Figure 1B). Immunostaining with an isotype-matched control (mouse IgG1) for R-10G showed no pleura-specific signal (Supplementary Materials Figure S1). We then asked if R-10G-reactive glycans are *N*-linked or *O*-linked glycans. GlcNAc-containing fractions of mouse lung lobes were obtained with wheat germ agglutinin (WGA)-coated beads. A Western blot analysis of the bead-bound material yielded two bands with molecular weights of >270 kDa which were immunoreactive for R-10G. The intensities of these bands were not diminished by PNGase F pretreatment (Figure 1C), indicating that R-10G-reactive glycans are found in high-molecular-weight glycoproteins and that *O*-linked glycans modified with 6-sulfo di-LacNAc are components of these glycoproteins that are present at a high density in the pleural mesothelia of the lungs of adult mice.

**Figure 2:**
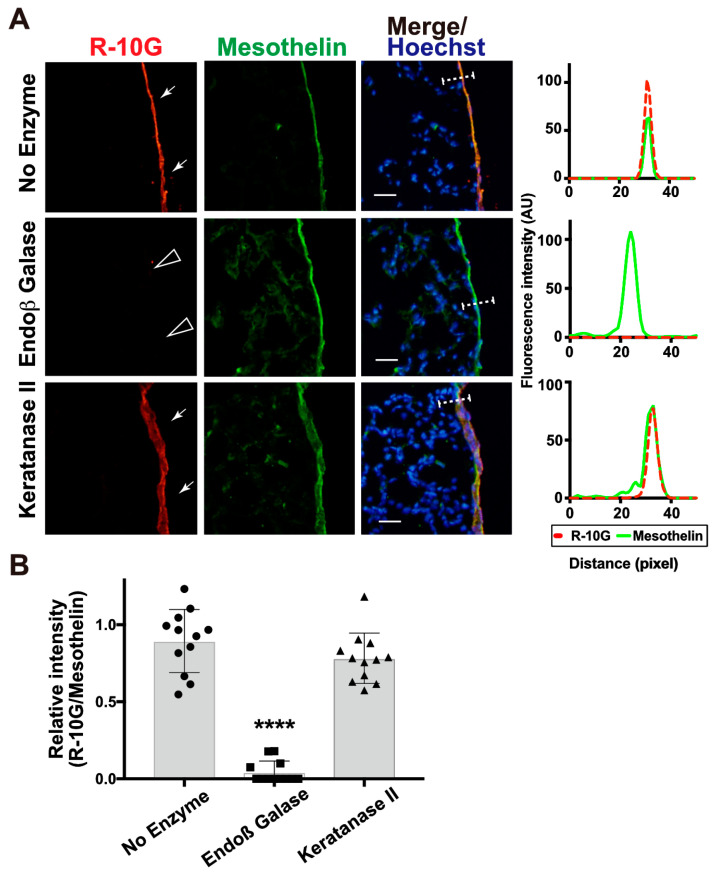
Endo-ß-galactosidase pretreatment diminishes R-10G signals abundant in the mouse pleural mesothelium. (**A**) Sections of lungs from normal adult mice were co-stained with R-10G (red) and anti-mesothelin (green) followed by Hoechst 33342 nuclear staining (blue). Sections were pretreated with a buffer only (no enzyme), Endo-β-galactosidase (Endoβ Galase), or Keratanase II, an endo-β-*N*-acetylglucosaminidase. Representative fluorescence microscopy images of the lower and central portions of the left lung lobe are shown (*n* = 3 per treatment). Dense R-10G staining signals in the pleural mesothelium (arrows) revealed by co-staining with an anti-mesothelin are demonstrated. Sections pretreated with Endo-β-galactosidase showed negligible levels of R-10G signals in the mesothelium (open arrowheads). Plot profiles of R-10G and mesothelin staining are presented. Signal intensities along the line marker (white dashed line) paths in the merged images were determined. (**B**) The relative intensity of R-10G to mesothelin is indicated (*n* = 12 mesothelia per treatment). Data were obtained from two experiments in which four pleural mesothelia from the lung specimens of three donors were analyzed for each treatment. **** *p* < 0.0001. Scale bar: 20 µm.

We then investigated whether the enzymatic removal of GlcNAc-6-sulfated or non-sulfated poly-LacNAc [42,43] could abolish R-10G immunoreactivity in the pleura. The pretreatment of lung sections with endo-β-galactosidase could abolish R-10G immunoreactivity (Figure 2A,B). This is consistent with the fact that R-10G epitope requires the 6-sulfo di-LacNAc structure as the minimum epitope structure for its recognition [5]. Anti-mesothelin signals arose from the mesothelin core protein since these signals were retained after treatment with endo-β-galactosidase (Figure 2A). Next, we wished to determine whether the R10G-reactive glycans were elongated from the repeated structures of GlcNAc-6-sulfated and Gal-6-sulfated or non-sulfated disaccharides. The pretreatment of lung sections with keratanase II, which cleaves the sulfated *N*-acetylglucosaminic β1-3 linkage to galactose in the non-reducing terminal chain, showed a level of R-10G immunoreactivity comparable to those in an non-enzyme-treated control (Figure 2A,B). These indicate that R-10G-glycans may be rather short and composed of two LacNAcs with GlcNAc-6-sulfation in the non-reducing terminal ends of glycans.

### 2.2. GlcNAc6ST2 Is Required and Sufficient for the Synthesis of R10G-Reactive KS Glycans in the Mouse Pleural Mesothelium

**Figure 3:**
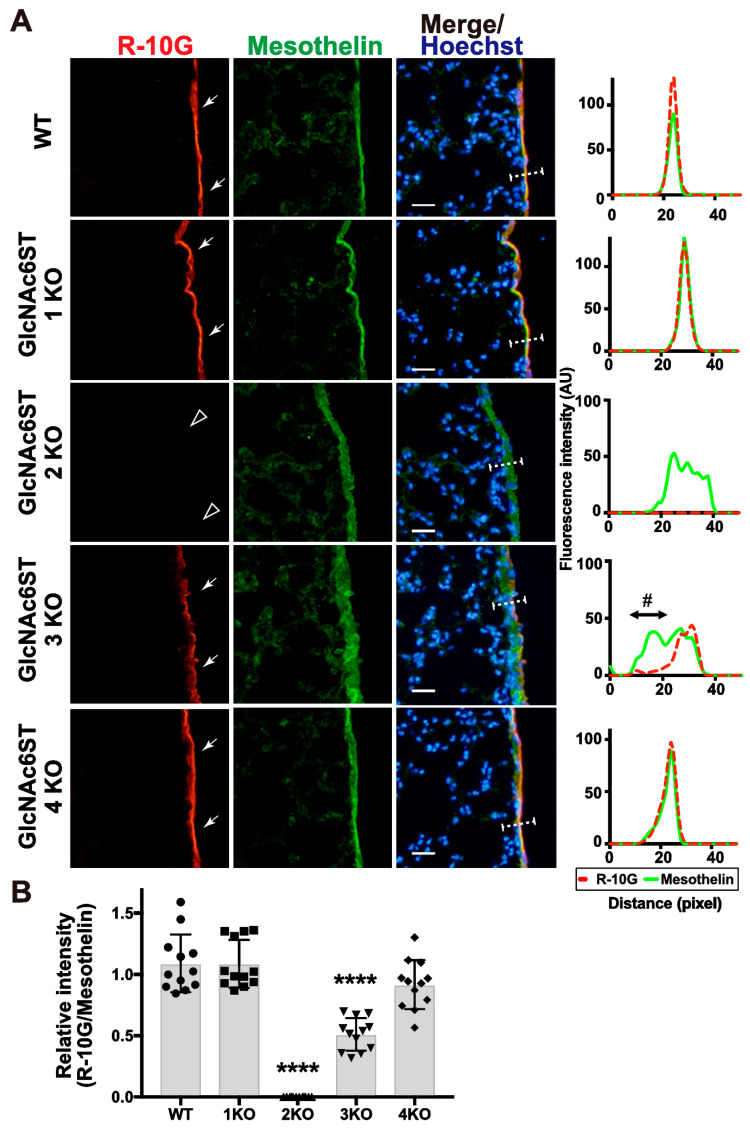
GlcNAc6ST2 is required for synthesis of R-10G-reactive sulfated glycans in the mouse pleural mesothelium. (**A**) Sections of lungs from wild-type (WT), *Chst2*-deficient (GlcNAc6ST1 KO) [14,15], *Chst4*-deficient (GlcNAc6ST2 KO) [15,21], *Chst5*-deficient (GlcNAc6ST3 KO) [24], and *Chst7*-deficeint (GlcNAc6ST4 KO) mice [9] were co-stained with R-10G (red) and anti-mesothelin (green), followed by Hoechst 33342 nuclear staining (blue). Dense R-10G staining in the pleural mesothelium is demonstrated (arrows). Sections of GlcNAc6ST2 KO showed negligible levels of R-10G signals in the mesothelium (open arrowheads), and GlcNAc6ST3 KO mice showed reduced levels of R-10G signals. The area proximal to the basement membrane structure in the mesothelial cell layer showed a selective reduction in R-10G immunoreactivity (#). Plot profiles of R-10G and mesothelin staining are presented. Signal intensities along the line marker (white dashed line) paths in the merged images were determined (*n* = 3 per genotype). (**B**) The relative intensity of R-10G to mesothelin is indicated (*n* = 12 mesothelia per genotype). Data were obtained from three experiments in which four pleural mesothelia from lung specimens from three donors were analyzed for each genotype. **** *p* < 0.0001. Scale bar: 20 µm.

We previously showed that the 6-sulfo sLe^X^ present in the high endothelial venule (HEV) cells of peripheral lymph nodes is complementarily synthesized by GlcNAc6ST1 and GlcNAc6ST2 [15,16]. Recently, we reported that GlcNAc6ST2 and GlcNAc6ST3 are complementary to the synthesis of sialyl 6-sulfo LacNAc recognized by CL40 in the mouse pleura [3]. We wished to determine which GlcNAc6ST is responsible for the synthesis of R10G-reactive glycans in the mouse pleural mesothelium. We expected that GlcNAc6ST3 might be a major GlcNAc6ST for pleural R-10G glycans since GlcNAc6ST3 is responsible for R-10G-reactive KS glycans in the brain [9]. Mice genetically deficient in each GlcNAc6ST gene were used for an analysis. Mice deficient in GlcNAc6ST1 or GlcNAc6ST4 showed a level of R-10G reactivity comparable to that of wild-type (WT) mice. GlcNAc6ST3-deficient mice showed about 50% reduced immunoreactivity. This reduction was selectively seen in the area proximal to the basement membrane structure of the mesothelial cell layer. Unexpectedly, mice deficient in GlcNAc6ST2 showed negligible levels of pleural R-10G signals (Figure 3A,B). As seen in the previous report [3], the mesothelin-positive mesothelium was thickened in GlcNAc6ST2 KO and GlcNAc6ST3 KO mice (Figure 3A). R-10G signals in these KO mice were not colocalized with laminins (Supplementary Materials Figure S2). These results indicate that both GlcNAc6ST2 and GlcNAc6ST3 are involved in the synthesis of R10G-reactive sulfated glycans in the mouse pleural mesothelium and that while GlcNAc6ST3 is partially involved, GlcNAc6ST2 is essential for synthesis. In these KO mice, the mesothelial cell layer may be distorted in the area proximal to the basement membrane for unknown reasons.

**Figure 4:**
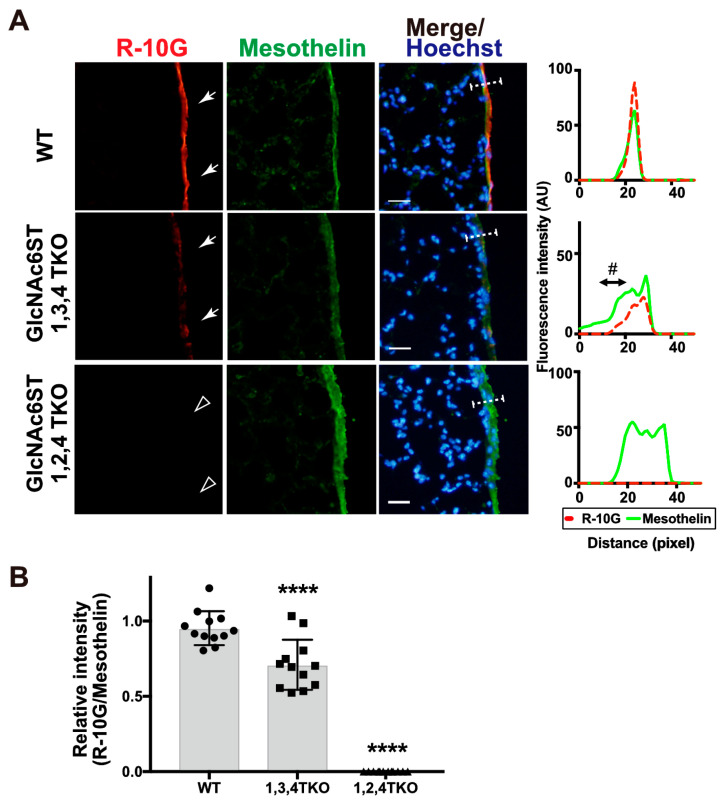
GlcNAc6ST1,2,4 triple-KO mice lack R-10G-reactive sulfated glycans in the pleural mesothelium. (**A**) Lung sections prepared from normal wild-type (WT), *Chst2/Chst5/Chst7* triple-deficient (GlcNAc6ST1,3,4 TKO), and *Chst2/Chst4/Chst7* triple-deficient (GlcNAc6ST1,2,4 TKO) [28] mice were co-stained with R-10G (red) and an anti-mesothelin (green), followed by Hoechst 33342 nuclear staining (blue). Dense R-10G staining in the pleural mesothelium is demonstrated (arrows). GlcNAc6ST1,2,4 TKO mice showed negligible levels of R-10G signals in the mesothelium (open arrowheads). Plot profiles of R-10G and mesothelin staining are presented. Signal intensities along the line marker (white dashed line) paths in the merged images were determined (*n* = 3 per genotype). The area proximal to the basement membrane structure in the mesothelial cell layer showed a selective reduction in R-10G immunoreactivity (#). (**B**) The relative intensity of R-10G to mesothelin is indicated (*n* = 12 mesothelia per genotype). Data were obtained from three experiments in which four pleural mesothelia from lung specimens from three donors were analyzed for each genotype. **** *p* < 0.0001. Scale bar: 20 µm.

We then tested R-10G immunoreactivity in mice triple-deficient (TKO) in GlcNAc6ST1, 3, and 4 but sufficient in GlcNAc6ST2 and mice triple-deficient in GlcNAc6ST1, 2, and 4 but sufficient in GlcNAc6ST3. In agreement with the results of the single-KO mice presented above, GlcNAc6ST1,3,4 TKO mice were found to have a significantly reduced level (~30%) of R-10G immunoreactivity. The GlcNAc6ST1,2,4 TKO mice showed a negligible level of mesothelial R-10G immunoreactivity (Figure 4A,B), indicating that GlcNAc6ST2 is essential for the synthesis of R-10G KS/KS-related glycans and that GlcNAc6ST3 has a partial role in their synthesis. These R-10G signals were not colocalized with laminins as seen in single-KO lungs (Supplementary Materials Figure S3).

**Figure 5:**
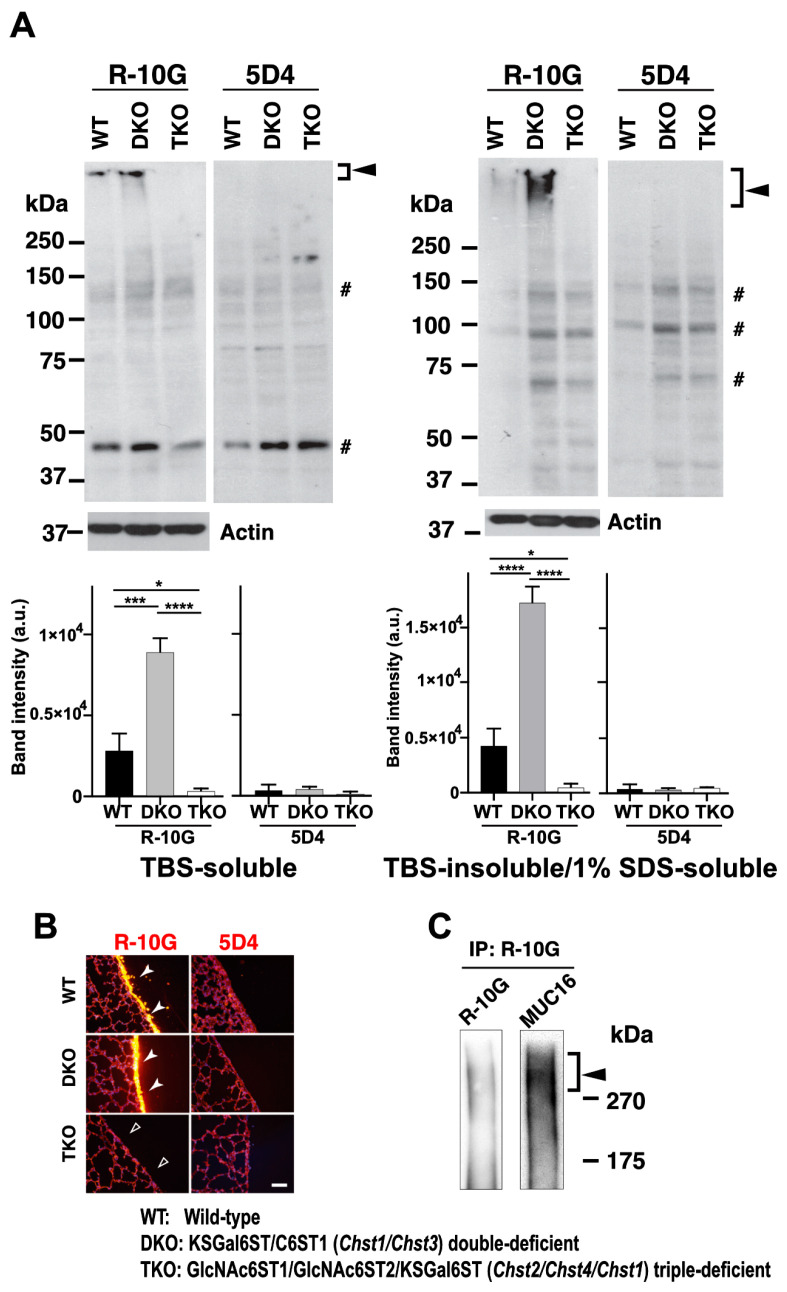
R-10G-immunoreactivity is augmented in the lungs of mice double-deficient in KSGal6ST and C6ST1, and MUC16 is present in R-10G-immunoprecipitated materials. (**A**) TBS-soluble fractions and TBS-insoluble/1% SDS-soluble fractions of lung tissues from normal wild-type (WT), *Chst1/Chst3* double-deficient (KSGal6ST/C6ST1 DKO), and *Chst2/Chst4/Chst1* triple-deficient (GlcNAc6ST1,2, and KSGal6ST TKO) [11,35] mice were prepared. A Western blot analysis was performed with R-10G and 5D4. R-10G-reactive bands with molecular weights of >250 kDa were observed in the WT and DKO (closed arrowheads) samples. Bands also seen in IgG_1_ control blots were indicated (*#*). Note that the intensities of the R-10G-immunoreactive bands in the DKO TBS-soluble and TBS-insoluble/1% SDS-soluble fractions are higher than those in the WT (*n* = 3 per genotype) samples. Data are presented as means ± SDs. * *p* < 0.05, *** *p* < 0.001, **** *p* < 0.0001. (**B**) Lung sections prepared from WT, KSGal6ST/C6ST1 DKO, and GlcNAc6ST1,2, and KSGal6ST TKO mice were stained with R-10G (left, orange) and 5D4 (right), followed by Hoechst 33342 staining (blue). Dense R-10G staining in the pleural mesothelium is shown (white arrowheads). The TKO mice showed negligible levels of R-10G signals in the mesothelium (open arrowheads). Scale bar: 25 µm. (**C**) The lung lysates of WT mice were used to prepare R-10G-immunoprecipitated (IP) materials as described in the Materials and Methods. IP materials were blotted with R-10G or an anti-MUC16. Smear bands with molecular weights of >270 kDa were observed (closed arrowhead). MUC16 was co-precipitated with R-10G-reactive 6-sulfo di-LacNAc.

KSGal6ST and C6ST1 can catalyze the sulfation modification to the 6-position of Gal on KS and related glycans [32,35,36,40]. We then asked if a double deficiency in these Gal-6-sulfotransferases would change the synthesis and localization of pleural R-10G glycans. We investigated R-10G immunoreactivity in KSGal6ST and C6ST1 DKO mice and GlcNAc6ST1, 2, and KSGal6ST TKO mice [11,35]. A Western blotting analysis with R-10G showed bands of molecular weights of >250 kDa in TBS-soluble fractions (Figure 5A left) and lower smear bands of molecular weight of >250 kDa in TBS-insoluble/1% SDS-soluble fractions (Figure 5A right) of lung lysates prepared from WT and DKO mice, whereas these high-molecular-weight band signals were not observed for the 5D4 anti-Gal-6S GlcNAc-6S-KS antibody [44] in either fraction of WT or DKO lungs.

Interestingly, the DKO samples showed a three- to fourfold increase in R-10G band intensity compared to the WT control. In the GlcNAc6ST1, 2, and KSGal6ST TKO samples, these high-molecular-weight band signals were not observed in either of the two fractions. Therefore, di-LacNAc structures with sulfation modifications on both Gal-6 and GlcNAc-6 may not intrinsically exist in R-10G-reactive glycans. In the lung sections of DKO mice, we observed enhanced R-10G immunoreactivity in the pleurae compared to WT mice (Figure 5B left). We did not observe the R-10G signal in GlcNAc6ST1, 2, and KSGal6ST TKO lung sections. 5D4-immunoreactivity in the mouse pleural mesothelium in all genotypes was not observed (Figure 5B right). The scRNA-seq data [45] showed high, selective expression of *Chst4*, *Msln*, and *Muc16* in mesothelial cells (Supplementary Materials Figure S4). The *Muc16* gene encodes Mucin-16 (MUC16), a highly *O*-glycosylated membrane-associated mucin. MUC16 can be extracellularly released by proteolytic cleavage. We tested if MUC16 is a carrier protein of 6-sulfo di-LacNAc recognized by R-10G. MUC16 was co-immunoprecipitated with R-10G in mouse lung lysates (Figure 5C), indicating that MUC16 could be a candidate molecule carrying R-10G-reactive glycans. R-10G and the anti-MUC16 antibody used were verified in OVCAR-3 cells [46,47] (Supplementary Materials Figure S5).

## 3. Discussion

Recently, we reported that sialyl 6-sulfo LacNAc is complementarily synthesized by GlcNAc6ST2 and GlcNAc6ST3 in the mouse pleura. Here, we show that R-10G-reactive KS/KS-related sulfated glycans are also present in the pleural mesothelium and that R-10G glycans are synthesized essentially by GlcNAc6ST2 in the mouse pleura. This is the first demonstration, as far as we know, that GlcNAc6ST2 is an enzyme that synthesizes R-10G-reactive KS/KS-related glycans in vivo.

**Figure 6:**
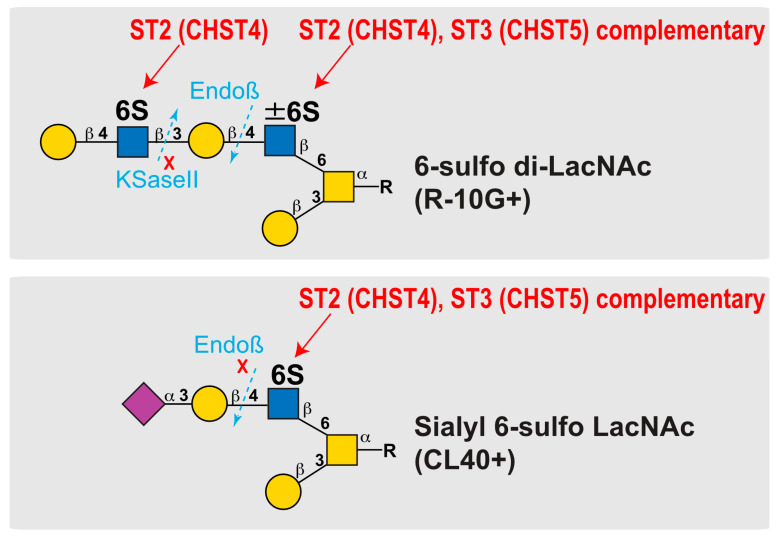
Schematic representations of the reaction modes of GlcNAc6ST2 and GlcNAc6ST3 to 6-sulfo di-LacNAc and sialyl 6-sulfo LacNAc. The modes of synthesis mediated by GlcNAc6ST2 (abbreviated ST2, also known as CHST4) and GlcNAc6ST3 (abbreviated ST3, also known as CHST5) are shown. Predicted susceptibilities to Endo-β-galactosidase (Endoβ) [43] and keratanase II (KSaseII) [50] in pleural *O*-glycans with asialo 6-sulfo di-LacNAc (Galβ1–4GlcNAc(6S)β1–3Galβ1–4GlcNAc(±6S)) or sialyl 6-sulfo LacNAc (Neu5Acα2-3Galβ1-4(6S)GlcNAc) [3] abundant in normal lungs are presented. Symbols denote the following: C-6 sulfate (S), galactose (Gal; yellow circle), *N*-acetylglucosamine (GlcNAc; blue square), *N*-acetylgalactosamine (GalNAc; yellow square), and *N*-acetylneuraminic acid, predominant sialic acid (Neu5Ac; purple diamond).

A possible explanation for the 50% reduction in R-10G recognition in the GlcNAc6ST3 KO mesothelium is the partial removal of sulfate groups in the R-10G recognition epitope. As we proposed and summarized (Figure 6), there could be a variation in the GlcNAc-6-sulfation of di-LacNAc in the lung pleura. The GlcNAc-6-sulfation of the penultimate LacNAc may not be essential for R-10G antibody recognition [5,6]. However, in the absence of this GlcNAc-6-sulfate, R-10G recognition would be significantly reduced. GlcNAc-6-sulfation of this penultimate LacNAc may be mediated by the complementary actions of GlcNAc6ST2 and GlcNAc6ST3, whereas the GlcNAc-6-sulfation of the non-reducing terminal LacNAc, which is essential for R-10G recognition, may be catalyzed by GlcNAc6ST2 alone (Figure 6). This substrate specificity of GlcNAc6ST2 may explain the R-10G immunostaining phenotype of GlcNAc6ST2 KO and GlcNAc6ST1,3,4 TKO mice. GlcNAc6ST3 utilizes core 2-branched GlcNAc as a better substrate in the pleura [48]. It is not known whether the glycosyltransferases involved in the synthesis of LacNAc repeats are dependent on this penultimate GlcNAc-6-sulfation. In mucin glycome analysis data, sulfated mono- or di-LacNAc is abundant in mucin glycans [49]. It is probable that an R-10G-positive glycan is a GlcNAc-6-sulfated dimeric LacNAc without sialic acids at the non-reducing terminal end. The CL40-reactive sialyl 6-sulfo LacNAc may be present in the distinct glycan chains (Figure 6). The extraction of sulfated glycan fractions from the lung tissues of various GlcNAc6ST-deficient mice and glycomic analyses of these samples will have a major impact on determining the synthetic pathways and structures of these sulfated glycans and glycan epitopes synthesized by GlcNAc6STs. These analyses are expected to be performed in the future. In addition, glycan microarray analysis may be a future approach to elucidating the presence or absence of diversity in the sulfated glycan structures recognized by R-10G and CL40.

KSGal6ST and C6ST1 can catalyze the Gal-6-sulfation of KS and related glycans in vivo [11]. 5D4 recognizes KS oligosaccharide structures with absolute dependence on both Gal-6- and GlcNAc-6-sulfation modifications [36,37]. Immunoreactivity of the 5D4 antibody was not observed in the lung lobes or the pleurae of mice under physiological conditions, as indicated by biochemical and histological studies. This suggests that KS and related glycans that contain LacNAc-repeating structures with both Gal-6-sulfation and GlcNAc-6-sulfation are absent or only present in very small amounts in the lung mesothelium. One possible reason for the increases in R-10G immunoreactivity in the KSGal6ST and C6ST1 DKO pleurae could be the elevated availability of adenosine 3′-phosphate 5′-phosphosulfate (PAPS; a sulfate donor) to GlcNAc6ST2 and GlcNAc6ST3 in the Golgi complex of mesothelial cells. This may have resulted in an enhanced GlcNAc-6-sulfation reaction and increased R-10G immunoreactivity. KSGal6ST and/or C6ST1 may be primarily involved in Gal-6-sulfation of other glycans in mouse mesothelial cells. These glycans may include 6′-sulfo sLex and sialyl 6′-sulfo LacNAc, which can be recognized by mouse sialic-acid-binding immunoglobulin-like lectin (Siglec)-F, a paralog of human Siglec-8 [38,51,52,53,54,55]. The mechanism of region-selective reductions in R-10G immunoreactivity in the GlcNAc6ST3 KO mesothelium is unknown. Whether R-10G-reactive sulfated molecules are different multiple proteins still remains to be determined. Whether GlcNAc6ST3 is specific to some protein species is an issue to be addressed. The possible relationship between sulfated mucins and proteoglycans in the airways and alveoli [11,56,57,58] and in the mesothelial cell layer in GlcNAc6ST3 deficiency is totally unknown. Because MUC16 binds highly to mesothelin [59,60] and the encoding gene, *Muc16*, is selectively expressed in mesothelial cells, as shown by scRNA-seq, MUC16 is a candidate R-10G-reactive sulfated molecule in mouse pleurae. A glycoproteomic analysis of pleural R-10G-reactive molecules would aid in identifying core proteins, as shown in a recent study of MUC16 prepared from OVCAR-3 cells [47]. The shed form of MUC16, known as CA125, promotes cell aggregation and binding to the peritoneal surface through its interaction with mesothelin [59,61]. The possible involvement of GlcNAc-6-sulfation in MUC16 proteolysis and its effect on mesothelin binding or on the recognition of other binding proteins [62] remain important issues for the future. The molecular function of MUC16 requires binding partners such as mesothelin, galectin 1, galectin-3, and E- and P-selectins [63]. These MUC16-mediated molecular interactions depend on *N*- or *O*-linked glycans [61,63,64]. Given that the R-10G epitope in the mouse pleura is predicted to be contained in *O*-linked glycans, our results support a role for the R-10G epitope in determining the interaction between MUC16 modified with R-10G-glycans and its binding partners. Furthermore, the glycosylation of MUC16/CA125 is known to differ between physiological and pathological conditions [65]. The regulation of R-10G epitope expression in physiological and disease states requires further elucidation. We found that multiple Hoechst-positive nuclei are abnormally layered in lung pleurae of GlcNAc6ST2 KO and GlcNAc6ST3 KO mice. These staining patterns were not observed in WT, GlcNAc6ST1 KO, or GlcNAc6ST4 KO mouse pleural mesothelia. Sulfation modification by GlcNAc6ST2 and GlcNAc6ST3 may play an important role in normal mesothelial layer formation and mesothelial cell proliferation and differentiation. The involvement of these enzymes and the genes encoding them in mesothelioma pathogenesis is an interesting topic of research [66]. Exploring the functions of sulfated glycans synthesized by these enzymes, including the R-10G and CL40 glycans, in disease states may lead to a better understanding of the pathogenesis of mesothelioma.

## 4. Materials and Methods

### 4.1. Antibodies and Enzymes

Materials were obtained commercially from the following sources: the R-10G anti-GlcNAc-6-sulfo KS antibody was purchased from Cosmo Bio (RIT-M001, Tokyo, Japan); the 5D4 anti-GlcNAc-6-, Gal-6-sulfo KS antibody (MABN2483), mouse anti-β actin (#A2228), and rabbit anti-laminin antibody (#L9393) were obtained from Sigma-Aldrich (St-Louis, MO, USA); the rabbit anti-mouse mesothelin antibody was obtained from IBL (28127, Fujioka, Japan); the mouse anti E-cadherin antibody was obtained from BD Bioscience (#610182, Franklin Lakes, NJ, USA); and normal mouse IgG_1_ was from Santa Cruz (#sc-3877, Dallas, TX, USA). The rabbit anti-MUC16 antibody was from LSBio (#LS-C754876, Shirley, MA, USA). The Cy3-conjugated goat anti-mouse IgG_1_ (#115-165-205), Alexa Fluor 488-conjugated goat anti-rabbit IgG (H+L) (#111-545-144), HRP-conjugated goat anti-mouse IgG_1_ (#115-035-205), HRP-conjugated goat anti-mouse IgG_2a_ (#115-035-206), and HRP-conjugated goat anti-rabbit IgG (H+L) (#111-035-144) were obtained from Jackson ImmunoResearch Laboratories (West Grove, PA, USA); Hoechst33342 was obtained from Dojindo (H342, Kumamoto, Japan). Peptide-*N*-glycosidase F (PNGase F; P0704S, *Flavobacterium meningosepticum*) was obtained from NEB (Ipswich, MA, USA). Endo-β-galactosidase (#100455, *Escherichia freundii*) and Keratanase II (#100812, *Bacillus* sp. Ks 36) were obtained from Seikagaku Corporation (Tokyo, Japan). The enzyme pretreatments were optimized previously [3,37].

### 4.2. Mice

GlcNAc6ST1-deficient (KO) [14,15], GlcNAc6ST2-KO [21], GlcNAc6ST3-KO [24], and GlcNAc6ST4-KO mice [9] were maintained on a C57BL/6J genetic background. GlcNAc6ST1,3,4 TKO mice and GlcNAc6ST1,2,4 TKO mice were generated as described previously [9,28]. KSGal6ST [35]/C6ST1 [41] DKO, and GlcNAc6ST1,2/KSGal6ST TKO mice were generated by cross-breeding the above KO mouse strains [11]. The genotyping of each KO mouse was carried out according to the original studies. The transcription levels of the *Chst2*, *Chst4*, *Chst5*, and *Chst7* genes in each KO lung were previously described [3]. Male and female mice aged 2–4 months were used in the experiments. All mice were maintained under controlled environmental conditions free of specific pathogens and provided with standard nutrition and water at the animal housing facilities of the authors’ institution. All experiments were approved by the Animal Research Committees of the authors’ institutions (Authorizations #3742, Univ Lille; #918, Wakayama Med Univ; and #21-094, Kansai Med Univ).

### 4.3. Mouse Tissues

The mice were anesthetized and transcardially perfused with phosphate-buffered saline (PBS). The lung lobes and tracheas were dissected; a phosphate-buffered solution (PB) containing 4% paraformaldehyde was injected through the airways. The left lung lobes were post-fixed overnight for cryo-sectioning in PB containing 4% paraformaldehyde, equilibrated with 30% sucrose in PBS, and embedded in an O.C.T. compound, Tissue-Tek (Sakura, Torrance, CA, USA). For use in a biochemical analysis, the lung tissues were snap-frozen after PBS perfusion and then stored at −80 °C.

### 4.4. Immunohistochemistry and Fluorescence Microscopy

Frozen lung tissue was cut into 10 µm thick sections in a cryostat and collected on MAS-coated glass slides (SF17293; Matsunami, Osaka, Japan). For pre-treatment, the sections were digested with 10 mU/mL endo-ß-galactosidase or 50 mU/mL Keratanase II in a 50 mM Tris–acetate buffer at a pH of 7.0 and at 37 °C for 24 h. The sections were stained with R-10G (10 µg/mL) and anti-mesothelin (1:200 dilution) as described previously [3]. Signals were acquired using a fluorescence microscope (BX41; Olympus, Tokyo, Japan), using the same exposure settings for each antibody staining. The fluorescence intensities of Cy3-R10G and Alexa Fluor 488–mesothelin in stained pleura in digital images were determined semi-quantitatively using ImageJ (NIH, Bethesda, MD, USA). Four pleural mesothelia per mouse were randomly selected. Three mice were tested for each genotype or treatment.

### 4.5. Immunoprecipitation

To carry out immunoprecipitation, 1% Triton X 100-soluble fractions were prepared from tissue homogenates of 100 mg lung lobes of 2- to 3-month-old C57BL/6J mice. The lung lysate was mixed with a complex of the R-10G anti-KS antibody and Protein G Dynabeads (Thermo Fisher Scientific, Waltham, MA, USA) in PB containing 0.02% Tween-20 (PB-T) for 30 min at room temperature. The immunocomplexes bound to the Protein G Dynabeads were isolated with the DynaMag-2 Magnet (Thermo Fisher Scientific).

### 4.6. WGA-Bead-Bound Precipitation

To carry out WGA-bead-bound precipitation, 1% Triton X 100-soluble fractions were prepared as described above. The fractions were incubated with GlcNAc-binding WGA-coated beads (Vector Laboratories, Newark, CA, USA) at 4 °C overnight. The bead-bound materials were used for a Western blot analysis.

### 4.7. Immunoblots

Frozen lung tissues were homogenized in Tris-buffered saline (TBS) with protease inhibitors as previously described [67]. The tubes were subsequently placed in a Bioruptor sonicator water bath (Cosmo Bio). The tissue was crushed 4–5 times for 15 s at maximum ultrasound power until no solids were visible in the tubes. The tissue was then ultracentrifuged at 100,000× *g* for 30 min at 4 °C. The supernatant fluid was collected (TBS-soluble fraction). The pellet was suspended in TBS containing 1% SDS, and the pellet was dissociated and centrifuged at 15,000× *g* for 20 min at room temperature. The supernatant fluid was collected (TBS-insoluble/1% SDS-soluble fraction). Immunoblotting was performed as described previously [37] with the following antibody concentrations: R-10G anti-KS (dilution 1:1000), 5D4 anti-KS (1:1000), anti-E-cadherin (1:1000), HRP-conjugated goat anti-mouse IgG1 secondary antibody (1:3000), goat anti-rabbit IgG (1:1000), and goat anti mouse IgG2a (1:5000).

### 4.8. Statistical Analysis

All data are presented as mean ± SE unless otherwise noted. The values were analyzed using a one-way analysis of variance with Dunnett’s test (vs. wild-type or without enzyme control) or Tukey’s test via Prism (GraphPad Software, La Jolla, CA, USA). *p*-values less than 0.05 were considered statistically significant.

## Figures and Tables

**Table 1:** The GlcNAc-6-*O*-sulfotransferase family and the Gal-6-*O*-sulfotransferase family.

Name	Other Nomenclature	Gene	Substrate	KS/Sulfated LacNAc Oligosaccharide Synthesis	Refs.
GlcNAc6ST1 *	*N*-acetylglucosamine 6-*O*-sulfotransferase (GlcNAc6ST); Carbohydrate sulfotransferase 2 (CHST2)	*CHST2*	GlcNAc	Yes	[8,12,13,14,15,16,17,18]
GlcNAc6ST2 *	High endothelial cell *N*-acetylglucosamine 6-*O*-sulfotransferase (HEC-GlcNAc6ST); L-Selectin ligand sulfotransferase (LSST); Carbohydrate sulfotransferase 4 (CHST4)	*CHST4*	GlcNAc	Yes (shown in this article)	[15,16,19,20,21]
GlcNAc6ST3	Intestinal *N*-acetylglucosamine 6-*O*-sulfotransferase (I-GlcNAc6ST); Carbohydrate sulfotransferase 5 (CHST5)	*CHST5* **	GlcNAc	Yes	[9,22,23,24]
GlcNAc6ST4	*N*-acetylglucosamine 6-*O*-sulfotransferase-4; Chondroitin 6-*O*-sulfotransferase-2 (C6ST-2); Carbohydrate sulfotransferase 7 (CHST7)	*CHST7*	GlcNAc, GalNAc	N.d. #	[9,25,26,27,28]
GlcNAc6ST5	Cornial *N*-acetylglucosamine 6-*O*-sulfotransferase (C-GlcNAc6ST); Carbohydrate sulfotransferase 6 (CHST6)	*CHST6* **	GlcNAc	Yes	[23,29,30,31]
KSGal6ST	Keratan sulfate galactose-6-*O*-sulfotransferase; Carbohydrate sulfotransferase 1 (CHST1)	*CHST1*	Gal	Yes	[19,32,33,34,35,36,37,38]
C6ST1	Chondroitin-6-*O*-sulfotransferase (C6ST); Carbohydrate sulfotransferase 3 (CHST3)	*CHST3*	GalNAc, Gal	Yes	[11,39,40,41]

* GlcNAc6ST1 and GlcNAc6ST2 are high-endothelial-venule-expressed sulfotransferases that are essential for the synthesis of L-selectin ligands [15,16]. ** *CHST6* and *CHST5* are genes that are homologous to each other. Primates have these two genes, while other mammalian genomes have only one of them [23,30]. # Abbreviations: N.d., not determined; GalNAc, *N*-acetylgalactosamine.

## Data Availability

The raw data supporting the conclusions of this article will be made available by the authors without undue reservation.

## Supplementary Materials

The following supporting information can be downloaded at https://www.mdpi.com/article/10.3390/molecules29040764/s1. Figure S1: Immunohistochemical analysis of mouse lung with control IgG_1_; Figure S2: Immunohistochemical analysis of the lung using R-10G and an anti-laminin antibody in single GlcNAc6ST-deficient mice; Figure S3: Immunohistochemical analysis of the lung using R-10G and an anti-laminin antibody in triple GlcNAc6ST-deficient mice; Figure S4: Single-cell RNA-seq data showing high, selective expression of *Chst4*, *Msln*, and *Muc16* in mesothelial cells in mouse lung; Figure S5: Immunoblotting analysis of R-10G and MUC16 in OVCAR-3 cells; supplemental data in Figure 5.
