# Supplementary material for: GlcNAc6ST2/CHST4 Is Essential for the Synthesis of R-10G-Reactive Keratan Sulfate/Sulfated N-Acetyllactosamine Oligosaccharides in Mouse Pleural Mesothelium
Source: Molecules. 2024 Feb 7;29(4):764. doi: 10.3390/molecules29040764 (PMC10893525; doi:10.3390/molecules29040764)
Supplement: Supplementary file 1 [file molecules-29-00764-s001.zip › molecules-2789560-supplementary.pdf]

Supplementary Materials

GlcNAc6ST2/CHST4 Is Essential for the Synthesis of R-10G-Reactive Keratan Sulfate/Sulfated *N*-Acetyllactosamine Oligosaccharides in Mouse Pleural Mesothelium

Yoshiko Takeda-Uchimura [1], Midori Ikezaki [2], Tomoya O. Akama [3], Yoshito Ihara [2], Fabrice Allain [1], Kazuchika Nishitsuji [1,2] and Kenji Uchimura [1,*]

[1] Univ. Lille, CNRS, UMR 8576—UGSF—Unité de Glycobiologie Structurale et Fonctionnelle, F—59000 Lille, France; yoshiko.uchimura@univ-lille.fr (Y.T.-U.); fabrice.allain@univ-lille.fr (F.A.); kazuchika.nishitsuji@univ-lille.fr or nishit@wakayama-med.ac.jp (K.N.)

[2] Department of Biochemistry, School of Medicine, Wakayama Medical University, Wakayama 641-8509, Japan; ikezaki@wakayama-med.ac.jp (M.I.); y-ihara@wakayama-med.ac.jp (Y.I.);

[3] Department of Pharmacology, Kansai Medical University, Osaka 570-8506, Japan; akamat@hirakata.kmu.ac.jp

* Correspondence: kenji.uchimura@univ-lille.fr ; Tel.:+33-(0)3-20-33-72-39

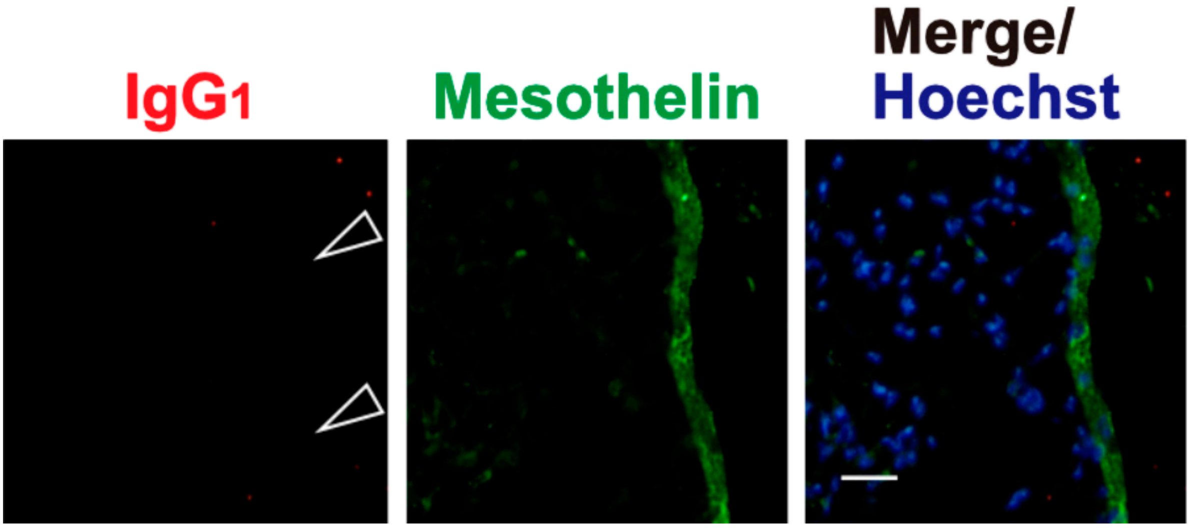

Figure S1. Immunohistochemical analysis of the mouse lung with control IgG_1. Lung sections prepared from normal wild-type mice were co-stained with control mouse IgG_1 (red) and an anti-mesothelin (green) followed by Hoechst 33342 nuclear staining (blue). Signals with control IgG_1 in the pleural mesothelium are negligible (open arrowheads) revealed by co-stained signals with a mesothelium marker, mesothelin. Representative fluorescence microscope images of the lower/middle region of left lung lobe are shown (n = 2). Scale bar: 20 µm.

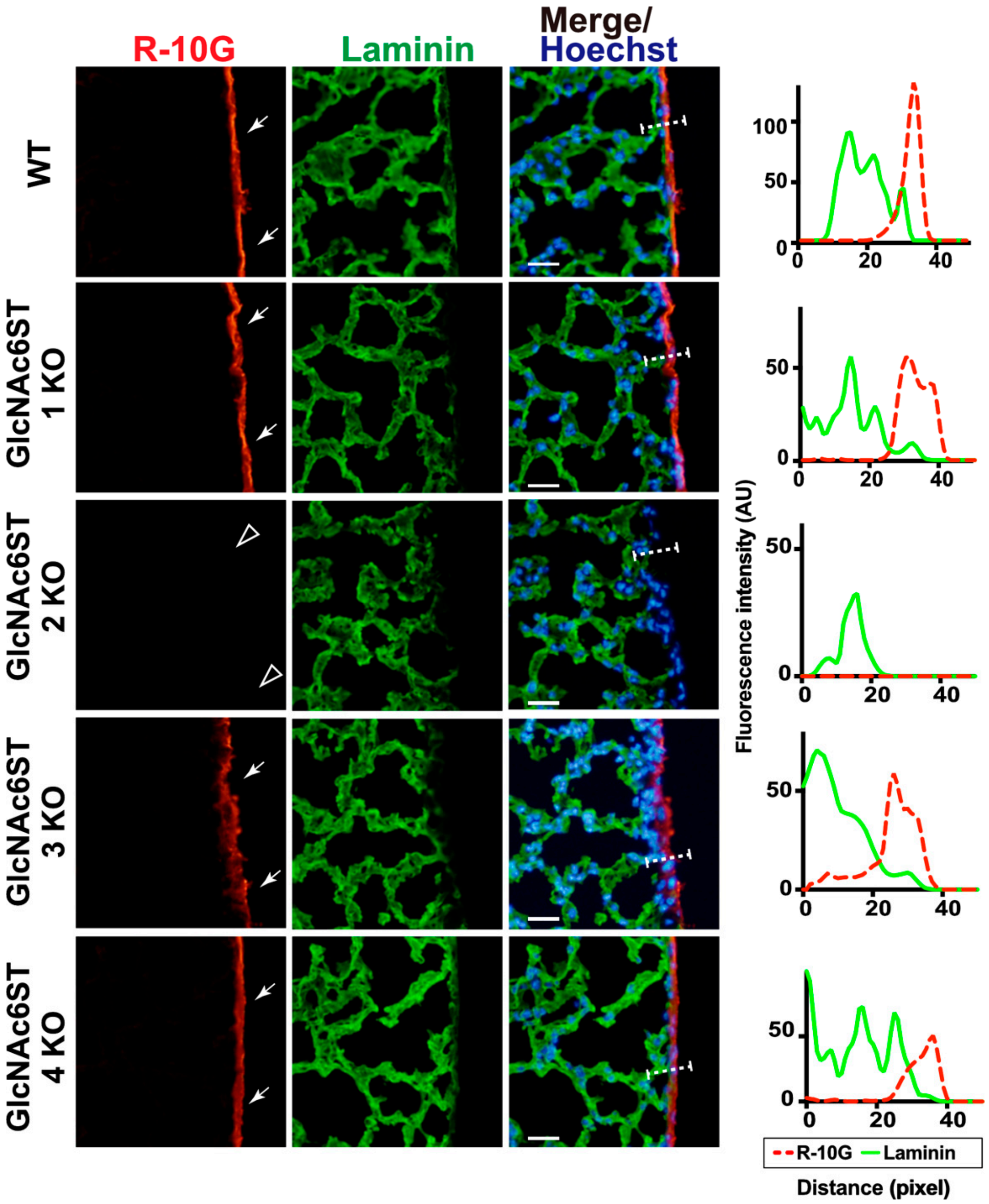

Figure S2. Immunohistochemical analysis of the lung using R-10G and an anti-laminin antibody in single GlcNAc6ST-deficient mice. Lung sections prepared from normal wild-type (WT), *Chst2*-deficient (GlcNAc6ST1 KO), *Chst4*-deficient (GlcNAc6ST2 KO), *Chst5*-deficient (GlcNAc6ST3 KO), or *Chst7*-deficeint (GlcNAc6ST4 KO) mice were co-stained with R-10G (red) and anti-laminin (green) followed by Hoechst 33342 nuclear staining (blue). Dense R-10G staining in the pleural mesothelium is shown (arrows). Sections of GlcNAc6ST2 KO showed negligible levels of R-10G signals in the mesothelium (open arrowheads). Digital images were captured using the same settings for each staining. The plot profiles of R-10G and laminin staining are shown. The signal intensities along the path of the line marker (dashed white line) in the merged images were measured as described in Materials and Methods (n = 3 for each genotype). Scale bar: 20 µm.

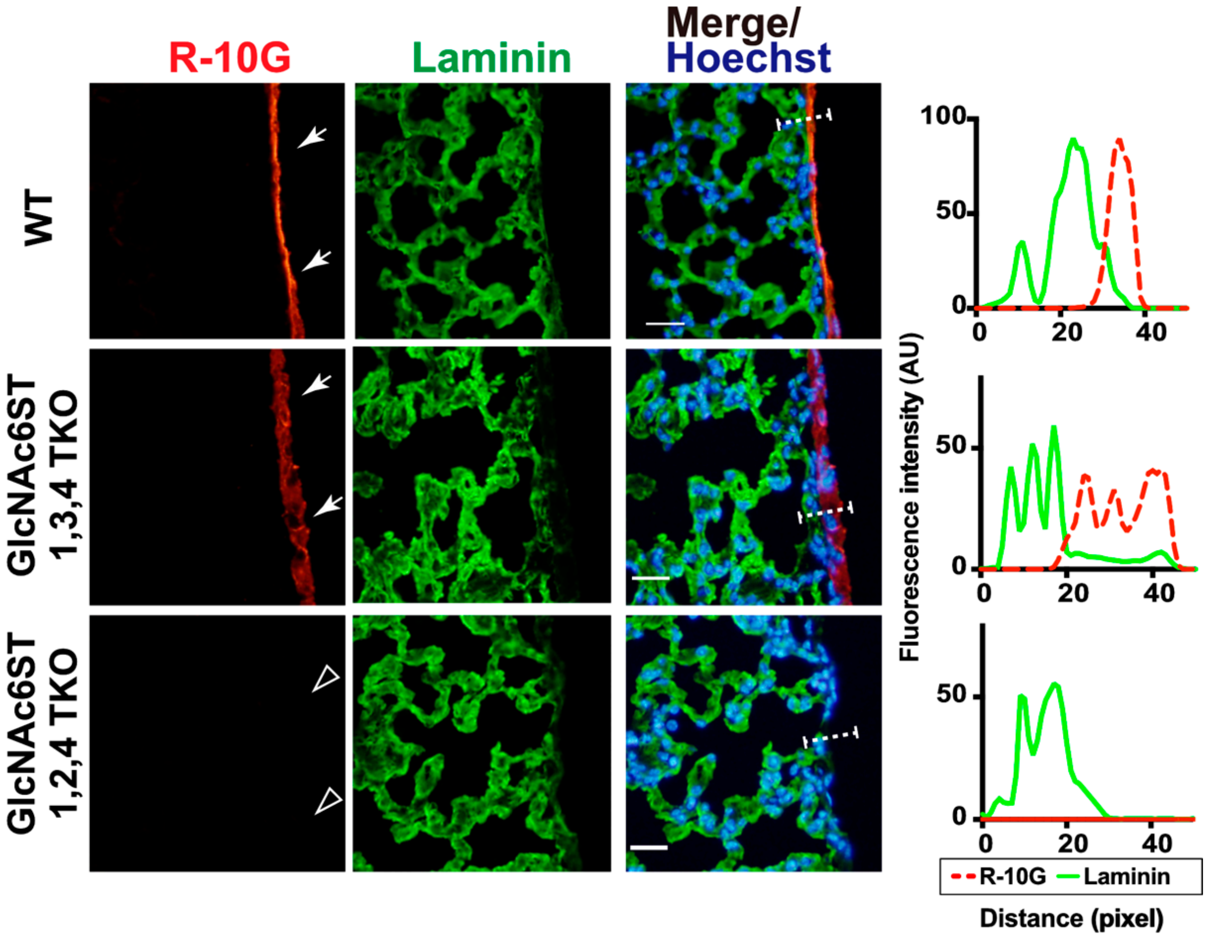

Figure S3. Immunohistochemical analysis of the lung using R-10G and an anti-laminin antibody in triple GlcNAc6ST-deficient mice. Lung sections prepared from normal wild-type (WT), *Chst2/Chst5/Chst7* triple-deficient (GlcNAc6ST1,3,4 TKO) and *Chst2/Chst4/Chst7* triple-deficient (GlcNAc6ST1,2,4 TKO) mice were co-stained with R-10G (red) and anti-laminin (green) followed by Hoechst 33342 nuclear staining (blue). Dense R-10G staining in the pleural mesothelium is shown (arrows). Sections of GlcNAc6ST1,2,4 TKO showed negligible levels of R-10G signals in the mesothelium (open arrowheads). Digital images were captured using the same settings for each staining. The plot profiles of R-10G and laminin staining are shown. The signal intensities along the path of the line marker (dashed white line) in the merged images were measured as described in Materials and Methods (n = 3 for each genotype). Scale bar: 20 µm.

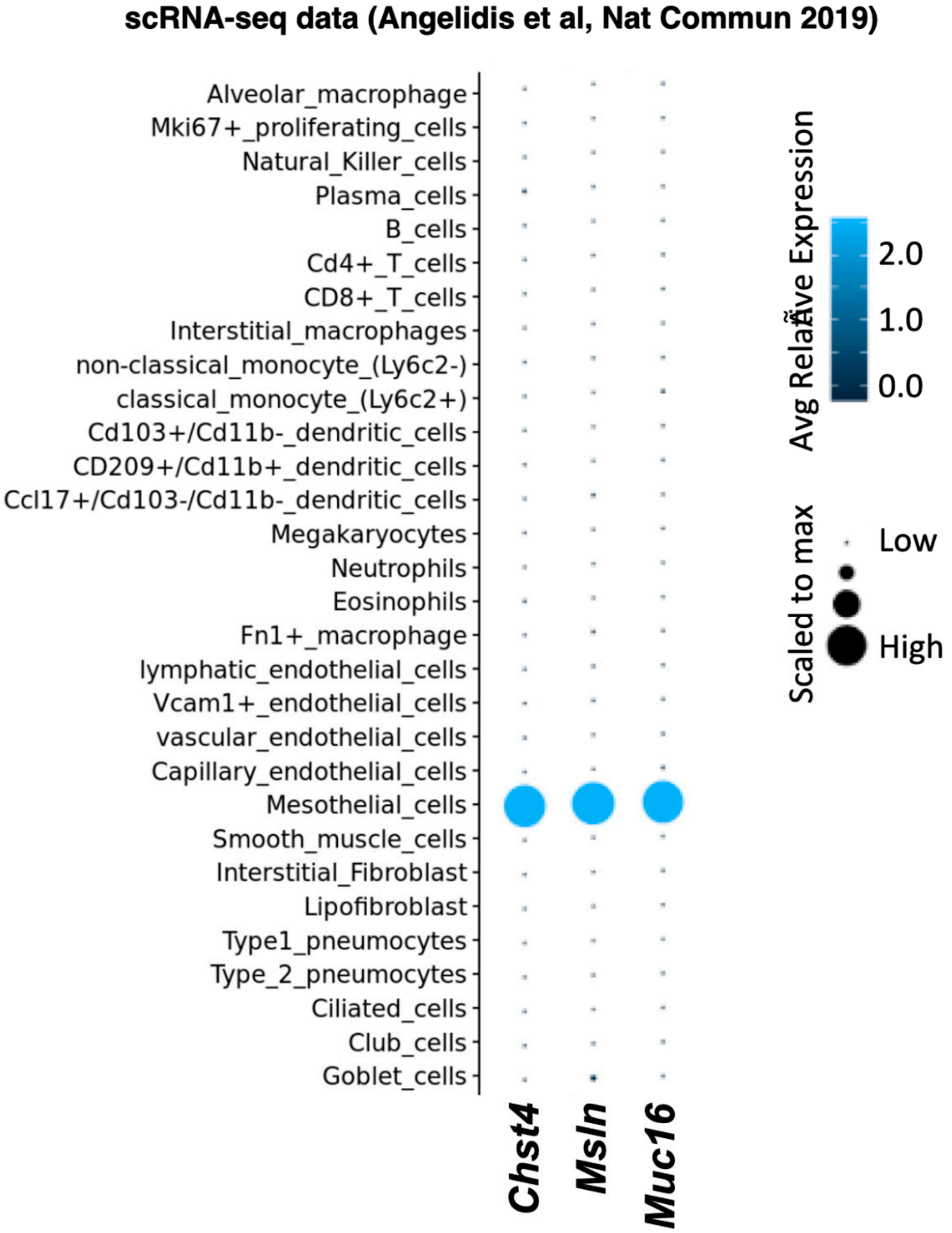

Figure S4. Single-cell RNA-seq data showing high, selective expression of *Chst4*, *Msln* and *Muc16* in mesothelial cells in mouse lung. Data pertinent to the genes of *Chst4, Msln and Muc16* were mined from a published single-cell RNA sequencing (scRNA-Seq) analysis of adult mouse lung (Angelidis, L. *et al.*, Nat Commun 2019, doi:10.1038/s41467-019-08831-9). Comparison of their transcription profiles in various cell types of the lung was performed using an scRNA-Seq transcriptome platform (https://theislab.github.io/LungAgingAtlas/).

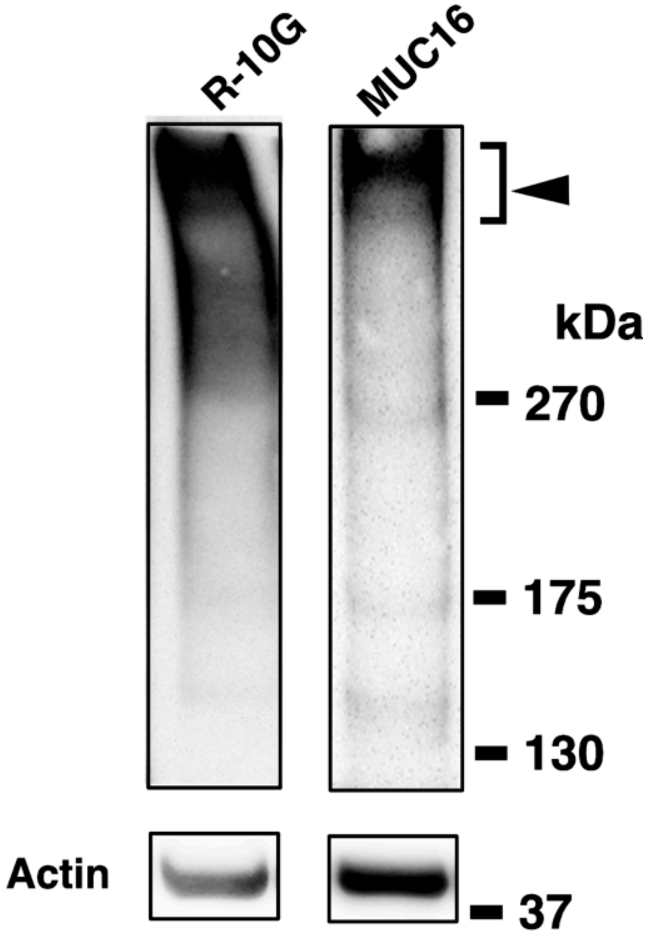

Figure S5. Immunoblotting analysis of R-10G and MUC16 in OVCAR-3 cells; supplemental data in Figure 5. OVCAR-3 cells express MUC16 that is post-translationally modified with core 1 and core 2 *O*-GalNAc glycans (Pedram K *et al*, Proc Natl Acad Sci USA. 2022, 119: e2117105119. doi:10.1073/pnas.2117105119). OVCAR-3 cells were cultured as previously described (Iwahashi N *et al*, Proc Natl Acad Sci USA. 2020, 117:33225-33234. doi: 10.1073/pnas.2009931117). Whole cell lysates of the cultured cells were prepared by means of 10% trichloroacetic acid precipitation. Western blotting was performed as described in Materials and Methods. Bands with molecular weights of > 270 kDa were observed (closed arrowhead). ß-Actin was used to show protein equal loading. Representative results are shown (n = 2). The validity of R-10G and the anti-MUC16 antibody used were verified.
